# The implementation of a blood conservation strategy in critical care on transfusion requirements

**DOI:** 10.1186/2197-425X-3-S1-A822

**Published:** 2015-10-01

**Authors:** A Tridente, E Smith, N Dempsey-Hibbert, S Bonney, P Nee

**Affiliations:** Whiston Hospital, Liverpool, United Kingdom; Manchester Metropolitan University, Manchester, United Kingdom

## Introduction

Patients admitted to intensive care units (ICUs) are commonly affected by anaemia, which is contributed to by iatrogenic blood loss from diagnostic testing [1], with potential risks of increasing transfusion requirements and negative effect on outcomes [2]. Recently published guidelines make several recommendations for the management of anaemia and red blood cells (RBCs) transfusion in adult ICU patients [3].

## Objectives

We aimed at determining whether a 3 components blood conservation strategy (BCS) can reduce blood volume drawn and/or RBCs transfusion requirements.

## Methods

The BCS consisted of 3 components:

1. Use of a HemoDraw blood conservation device at arterial or venous access to eliminate the need to discard blood prior to sampling
2. Use of paediatric sample tubes instead of adult ones for haematology tests, and half-filled adult sample containers for biochemistry tests
3. Raised awareness of the benefits of a restrictive transfusion policy on critical care.

The BCS was introduced in our ICU between 01/04/14 and 30/09/14 (period 2) for all adult level 3 critical care patients. A comparable retrospective control group of the same time period in 2013 was used (period 1).

Multiple linear regression analysis, adjusted for age, gender, APACHE II score and ICU length of stay (LOS), was used to assess the influence of the BCS on blood volume drawn. Logistic regression analysis was used to assess influence on likelihood of RBCs transfusion. STATA 10.1 (http://www.stata.com) was used for all analyses.

## Results

A total of 377 patients were recruited (198 for period 1, 179 for period 2), 208 (55.2%) were male. Median (IQR, interquartile range) age was 60 (45-73) years, median (IQR) APACHE II score was 17 (11-22), median (IQR) LOS was 3.7 (1.7-8.7) days. The median (IQR) blood volume drawn was 113.8 (48-252.1) ml for period 1 and 53.8 (18.8-111.2) ml for period 2. After correction for age, gender, APACHE II score, ICU LOS, the use of the BCS in period 2 was associated with a reduction of 88.5 ml (95% CI 71-105.9, p < 0.001) in blood drawn for diagnostic testing. There was no significant difference in likelihood of RBCs transfusion (OR 1.2, 95% CI 0.73-1.95, p = 0.49).

## Conclusions

Use of a BCS in critical care significantly reduced the volume of blood drawn for diagnostic testing during ICU stay, but a significant decrease in transfusion requirements could not be demonstrated.
